# Genomic epidemiology of SARS-CoV-2 from Uttar Pradesh, India

**DOI:** 10.1038/s41598-023-42065-6

**Published:** 2023-09-08

**Authors:** Gauri Misra, Ashrat Manzoor, Meenu Chopra, Archana Upadhyay, Amit Katiyar, Brij Bhushan, Anup Anvikar

**Affiliations:** 1 Molecular Diagnostics and COVID-19 Kit Testing Laboratory, National Institute of Biologicals (Ministry of Health and Family Welfare), A-32, Sector-62, Institutional Area, Noida, UP 201309 India; 2 National Dairy Research Institute, Karnal, Haryana India; 3 Bioinformatics Facility, Centralized Core Research Facility, All India Institute of Medical Sciences, Ansari Nagar, New Delhi, 110029 India

**Keywords:** Molecular biology, Diseases

## Abstract

The various strains and mutations of SARS-CoV-2 have been tracked using several forms of genomic classification systems. The present study reports high-throughput sequencing and analysis of 99 SARS-CoV-2 specimens from Western Uttar Pradesh using sequences obtained from the GISAID database, followed by phylogeny and clade classification. Phylogenetic analysis revealed that Omicron lineages BA-2-like (55.55%) followed by Delta lineage-B.1.617.2 (45.5%) were predominantly circulating in this area Signature substitution at positions S: N501Y, S: D614G, S: T478K, S: K417N, S: E484A, S: P681H, and S: S477N were commonly detected in the Omicron variant-BA-2-like, however S: D614G, S: L452R, S: P681R and S: D950N were confined to Delta variant-B.1.617.2. We have also identified three escape variants in the S gene at codon position 19 (T19I/R), 484 (E484A/Q), and 681 (P681R/H) during the fourth and fifth waves in India. Based on the phylogenetic diversification studies and similar changes in other lineages, our analysis revealed indications of convergent evolution as the virus adjusts to the shifting immunological profile of its human host. To the best of our knowledge, this study is an approach to comprehensively map the circulating SARS-CoV-2 strains from Western Uttar Pradesh using an integrated approach of whole genome sequencing and phylogenetic analysis. These findings will be extremely valuable in developing a structured approach toward pandemic preparedness and evidence-based intervention plans in the future.

## Introduction

The severe acute respiratory syndrome coronavirus 2 (SARS-CoV-2) is extremely contagious and has spread throughout the world, leading to over 593 million confirmed cases and 6.4 million deaths till August 21, 2022, globally, since its appearance in Hubei province, China, in December 2019^1^. India reported its first case on January 30, 2020, from Kerala^2^. As on August 21, 2022, India reported over 40 million SARS-CoV-2 cases with a 5.3 lakh death toll (WHO Coronavirus Disease (COVID-19) dashboard available online at: https://covid19.who.int/). The world has witnessed three outbreaks due to *coronaviruses* with significantly high morbidity rates which were: SARS-CoV in 2002, MERS-CoV (Middle East Respiratory Syndrome coronavirus) in 2012, and COVID-19 in 2019^3,4^. SARS-CoV-2 displays higher pathogenicity and transmissibility as compared to MERS-CoV and SARS-CoV^5,6^.

*Coronaviruses* are enveloped positive sense RNA viruses, ranging from 60 to 140 nm having an approximate genome size of 26–32 kb^7^. Belonging to the *Coronaviridae* family and genus *Betacoronavirus*, they are positive-sense single-stranded RNA genomes and encode for sixteen nonstructural (NSP1-NSP16) and four structural proteins (nucleocapsid, envelop, membrane, and spike glycoprotein). Spike (S) glycoprotein has a crown-like appearance that facilitates its attachment to the surface angiotensin-converting enzyme 2 (ACE2) receptor^8,9^. The receptor binding domain (RBD) which is 223 residues in length primarily binds to the ACE2 receptor^9,10^.

Beginning in the 1960s, patients with the common cold were the first group of people infected with human *coronaviruses* (HCoVs). Following this, seven HCoVs have been reported that infect humans: 229E, OC43, SARS-CoV, NL63, HKU1, MERS-CoV, and SARS-CoV-2^11–15^. SARS-CoV-2 is subjected to genetic evolution and subsequent antigenic variations through mutations, enabling them to synchronize in human hosts. This may impact the structure and functional activity of the virus^16^. Salient mutations specifically D614G were reported to be associated with viral transmissibility, high virulence, and less protection with current vaccines^17^. One of these variants was discovered in humans, and it was this version that spread the disease from an infected farmed mink in Denmark. However, it did not relate to the increased transmissibility^18,19^. The World Health Organization's (WHO) Technical Advisory Group on Virus Evolution classifies variations that pose an enhanced danger to global public health as "Variants of Concern" (VOC)^20^. In January 2021, WHO classified B.1.617.2 (Delta variant) as a VOC which had a Spike (S) double mutation namely E484Q and L452R. According to WHO guidelines, every reported variant is categorized as either a variant of concern (VOC) or a variant of interest (VOI)^21^. Thereafter, in April 2021, Delta was sub-classified to Delta sub-variants namely AY.1, AY.2, and AY.3 comprising of an additional Spike protein substitution K417N. The predominant variant reported in India in October 2020 was B.1.617 which was later observed in more than 20 countries. It contained two significant mutations (E484Q and L452R) in the sequence of protein S, and therefore was also known as a “double mutant”. The name was not rational due to the presence of 11 other mutations. Of the 11 mutations, the P681R mutation had the potential to induce greater pathogenicity with higher affinity towards the ACE2 receptor resulting in immune system evasion^20^. The second wave of the COVID-19 pandemic struck India adversely in 2021, along with numerous post-vaccination breakthrough infections brought on by novel strains^22^.

**Figure 1:**
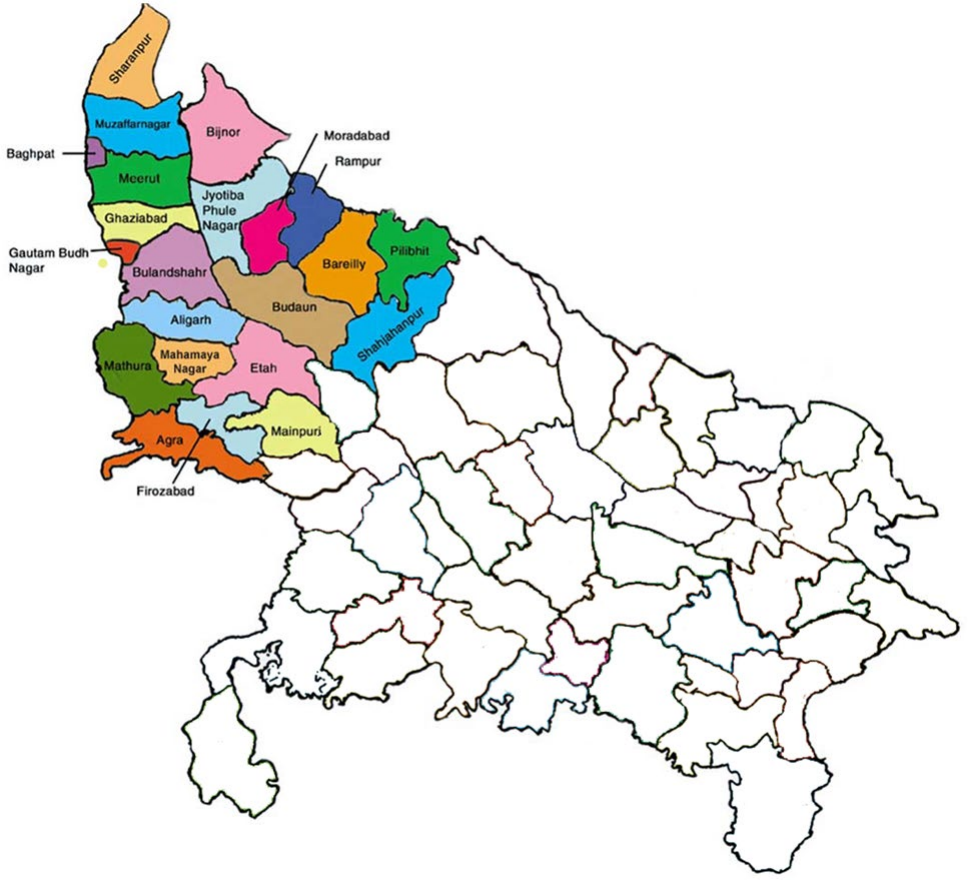
Highlighted regions of the map of Uttar Pradesh displaying the sample collection points.

India has witnessed an upsurge in the incidence of distinct SARS-CoV-2 strains in different states since the beginning of the pandemic. The clade I/A3i is a distinctive phylogenetic cluster identified from Indian genomes, dominated the early months of the pandemic (March and April), but by late April, a shift in clade frequency was seen, with most states revealing a higher presence of the Nextstrain clade A2a^23^. Uttar Pradesh is one of the states with the highest population densities in the nation (Census 2011, India) with an international trans-border with Nepal (Fig. 1). Cases proliferated more due to the travel from metro cities from March to April 2021 which gave rise to the deadly variants in the Western Uttar Pradesh region^24^. Basti, a district from Uttar Pradesh had reported the first instance of SARS-CoV-2 infection, which further lead to the spread of infection and transmission in the nearby areas of Uttar Pradesh^23^. Uttar Pradesh has been hit badly due to the second spike of the pandemic in 2021, in addition to many post-vaccination breakthrough infections due to new variants^25^. Our primary focus was on VOCs due to their major impact on public health.

Deployment of important functional bioinformatics tools like whole genome analysis of viral materials aids in the discovery of novel SARS-CoV-2 genetic variants spreading in populations, especially in the case of a global pandemic^26,27^. Genomic surveillance is an essential approach for investigating any outbreak and mapping the virus evolution and spread in case of emerging and re-emerging viruses and has resulted in the identification of variations with spike protein mutations that may confer increased transmissibility and immune evasion (such as alterations D614G, E484K/Q, K417T, N501Y, and P681H)^28–31^. Lineages have been used to define SARS-CoV-2 genetic variation, and an extensive nomenclature scheme has been devised^32^. During the SARS-CoV-2 pandemic, molecular and genetic characterization along with phylogenetic studies were exploited to investigate and monitor the dimensions of virus transmission^33–41^. In particular, large-scale or whole genome sequencing has culminated in the identification of genomic variants correlated with increased transmissibility, virulence, or evasion of host immune response across the world^42,43^. Tracking the emergence, dissemination, and genetic features of lineages, particularly variations of concern (VOCs), has become crucial due to their greater transmissibility, potential increased clinical severity, and ability to evade host immune responses^44^.

While an array of studies on the genetic epidemiology of SARS-CoV-2 from various Indian states have been published^23,41,45–47^, there has been a paucity of genomic data for SARS-CoV-2, particularly from Western Uttar Pradesh needed to assess the genetic epidemiology of COVID-19 and the prevalence of different lineages of the virus in the state. As a result, identifying and characterizing circulating lineages and sub-lineages is a cornerstone of worldwide epidemiological surveillance and policy^44^. All of these findings comprehensively underscore the critical significance of genomic monitoring programs in enabling countries to deploy evidence-based measures to combat the emergence and spread of novel SARS-CoV-2 variants^48^. The present study focuses on the molecular surveillance of SARS-CoV-2 strains from Western Uttar Pradesh to produce a robust database. This is the first comprehensive study from the Western region of Uttar Pradesh that recapitulates the SARS-CoV-2 variants prevalent in this region during the pandemic using molecular-based approaches.

## Results

### Whole genome sequence analysis

A total of 3,485 samples tested positive in real-time (RT-PCR) for SARS-CoV-2 in this study and 99 samples were processed for whole genome sequencing. The Ct values for positive tests ranged from 19 to 21 Ct which was in accordance with the WHO guidelines for clinical samples. Library preparation and quantification yielded 150 bp paired-end reads that were further diluted to final optimal loading concentrations for cluster amplification and sequencing.

### Clade distribution

**Figure 2:**
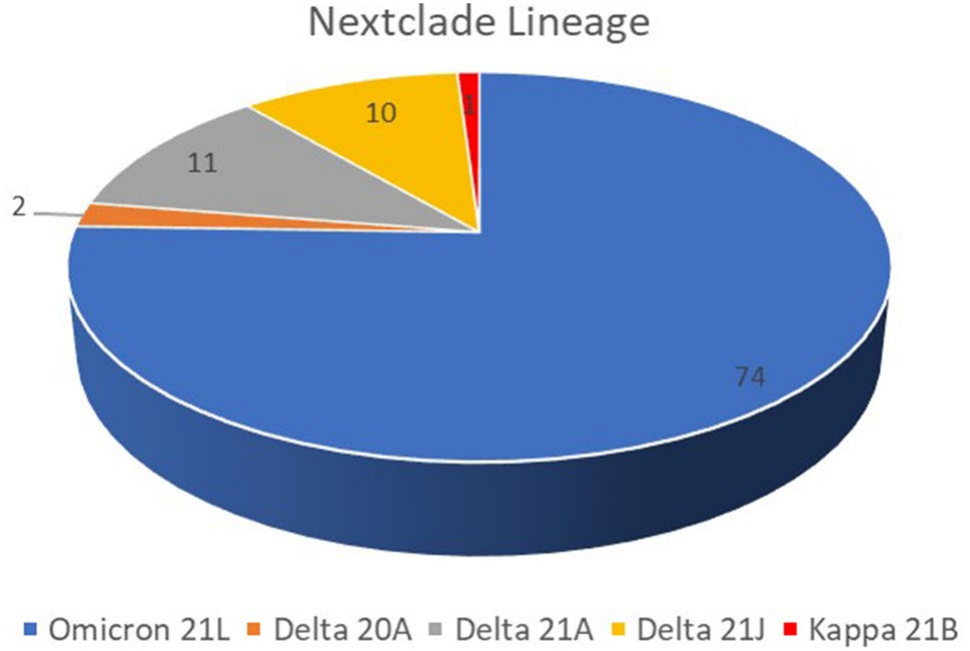
Next Clade classification among 99 genomic sequences of Delta and Omicron Variants.

**Figure 3:**
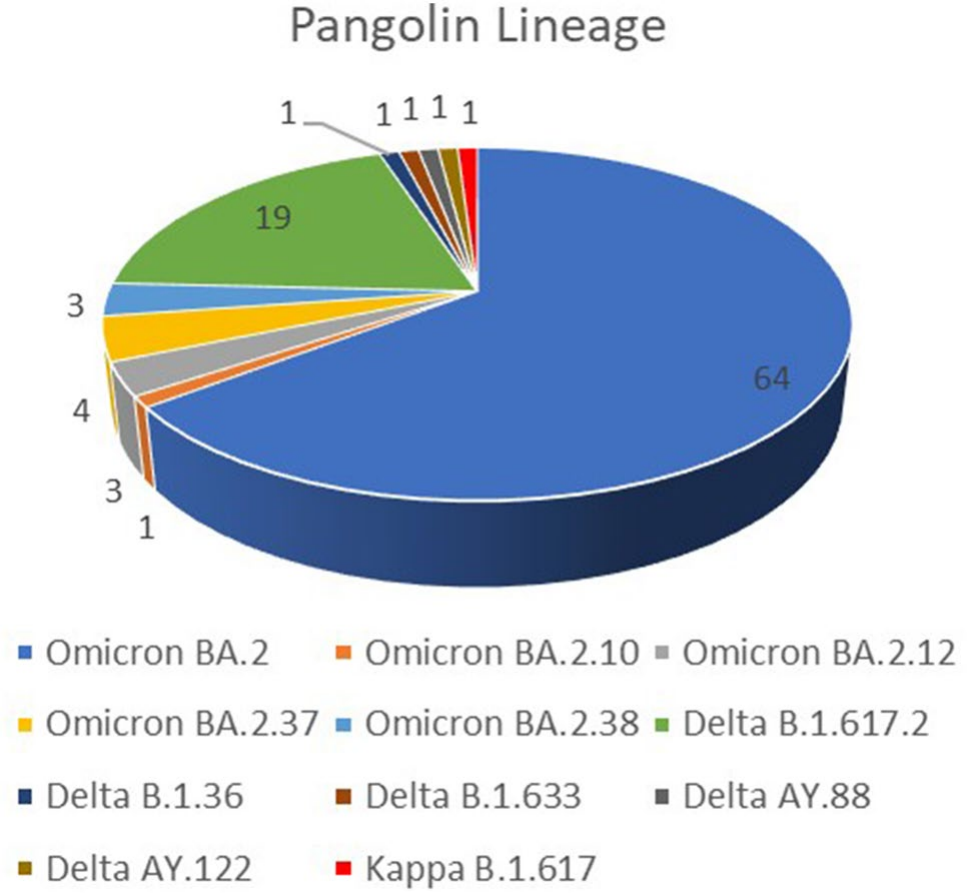
Pangolin classification among 99 genomic sequences of Delta and Omicron Variants.

From March 2021–Jan 2022, 99 SARS-CoV-2 sequences from Uttar Pradesh were analyzed using Pangolin (V4.1.3) and Next Clade (V2.9.1). The phylogenetic analysis confirmed that SARS-CoV-2 sequences were grouped into two major lineages, Delta (B.1.617.2-like) and Omicron (BA.2-like) (Supplementary File 1). As per the Next Clade classification, the outbreak showed six major clades of SARS-COV-2: 21L-Omicron (75.7%) followed by 21A (Delta) 11/99 (11.1%), 21J (Delta) 10/99 (10%), 20A 2/99 (2.02%) and 21B (Kappa) 1/99 (1%) of SARS-CoV-2 (Fig. 2). Further, according to the Pangolin lineage, VOCs were detected with a majority of the Omicron variant (BA.2-like: 75.7%) followed by the Delta variant (B.1.617.2: 19.19%, AY.122: 1%, AY.88: 1.01%, B.1.36: 1.01%, B.1.633: 1.01%) and kappa variant (B.1.617: 1.01%) as represented in Fig. 3. Using the Pangolin software's web version, the Pangolin lineage of the aforementioned sequences was discovered (https://Pangolin.cog-uk.io/).

### Distribution of mutations in the spike protein sequence

**Figure 4:**
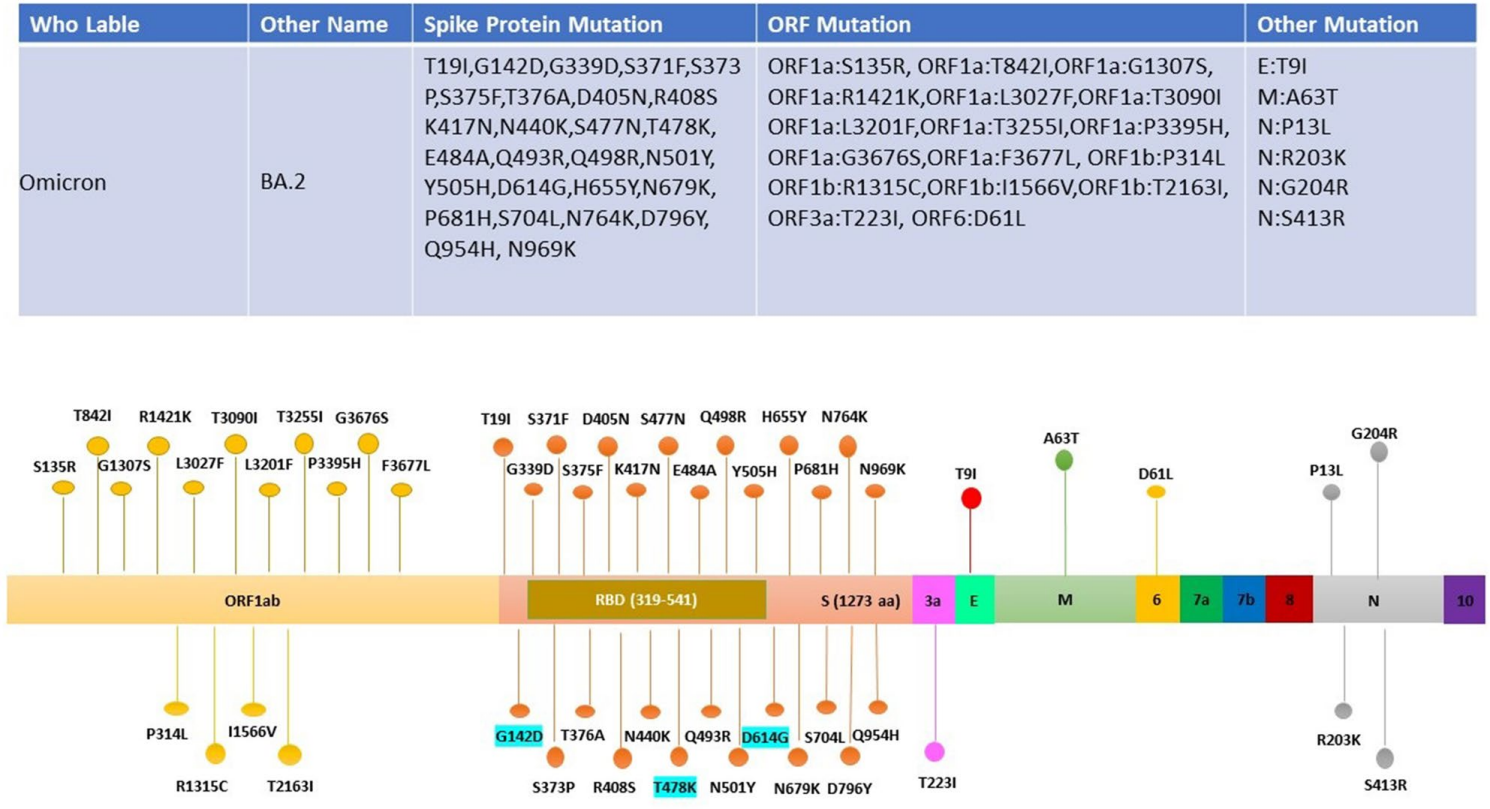
Representation of mutations observed within 75 samples of the Omicron Variants with different colors corresponding to different genes. Mutation common (G142D, T478K, and D614G) in Omicron and Delta variants are highlighted with blue color.

**Figure 5:**
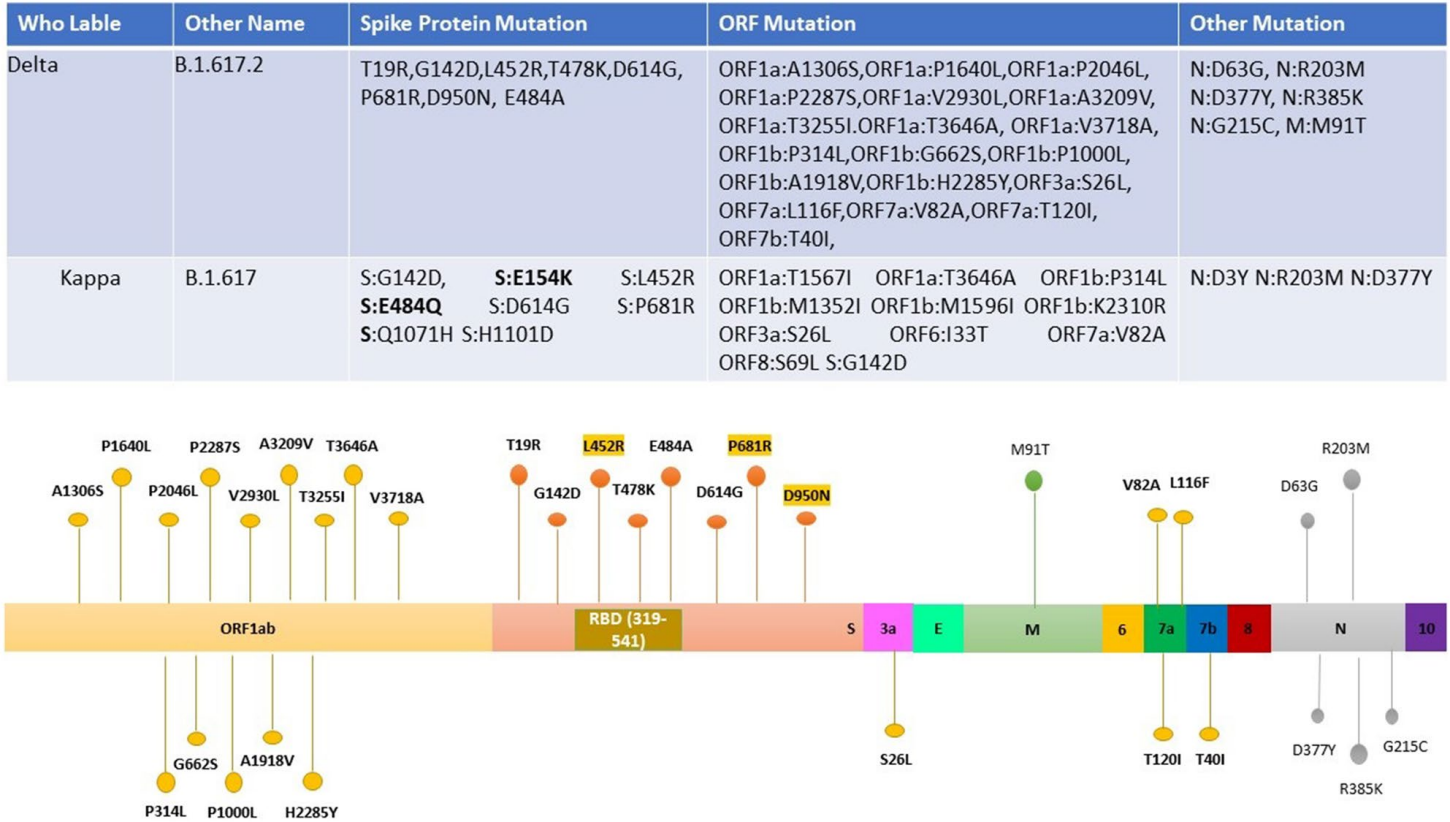
Representation of mutations observed within 23 samples of Delta Variant with different colors corresponding to different genes. Specific Mutations (L452R, P681R, and D950N) in the Spike protein of the Delta variants are highlighted with yellow color.

**Figure 6:**
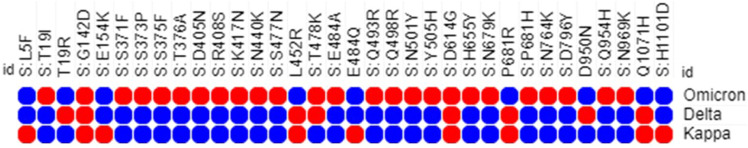
Heatmap of presence, and absence of spike protein mutation among 99 genome sequences. Red color represents the presence and blue represents the absence.

The presence of particular mutations is directly associated with the lineage assignment during the early pandemic period when SARS-CoV-2 genetic diversity is low. Genome analysis and study of the 99 SARS-CoV-2 genomic sequences demonstrated a total of 50 mutations in BA.2 (Omicron) and 33 in B1.167.2 (Delta) lineage as compared to a reference genome, across the genome (Supplementary File 2). Out of 50 mutations, 27 mutations were located in the spike protein of Omicron. Likewise, out of 34, seven were located in the S gene of the Delta variant (Figs. 4, 5). B.1.617.2 was initially identified in India in December 2020 and carries the spike mutation T19R, G142D, L452R, T478K, D614G, P681R, and D950N including two mutations in the NTD (T19R, G142D), two in the RBD (L452R and T478K), two mutations close to the furin-cleavage site (D614G, P681R), and one in the S2 region (D950N). Signature substitutions according to the total number of occurrences (B1.617.2 and BA.2) are D614G (266), S: T478K (261), S: G142D (165), S: N679K (150), S: P681H/R (152), S: H655Y (149), S: E484A (147), S: Q493R (147), S N501Y (145) while S: T478K (187), P681R (113) and L452R (112) are specifically present in B1.617.2 lineage (Delta variant). It is also noted that Kappa (B.1.617) carried additional mutations, namely S: E154K, and S: E484Q. Some additional mutations are also present in the Delta, Omicron, and Kappa variants, distinguishing them from each other (Fig. 6). These mutations are correlated to enhanced transmissibility, infectivity, receptor binding, and immune escape of the virus.

### Phylogenetic analysis

**Figure 7:**
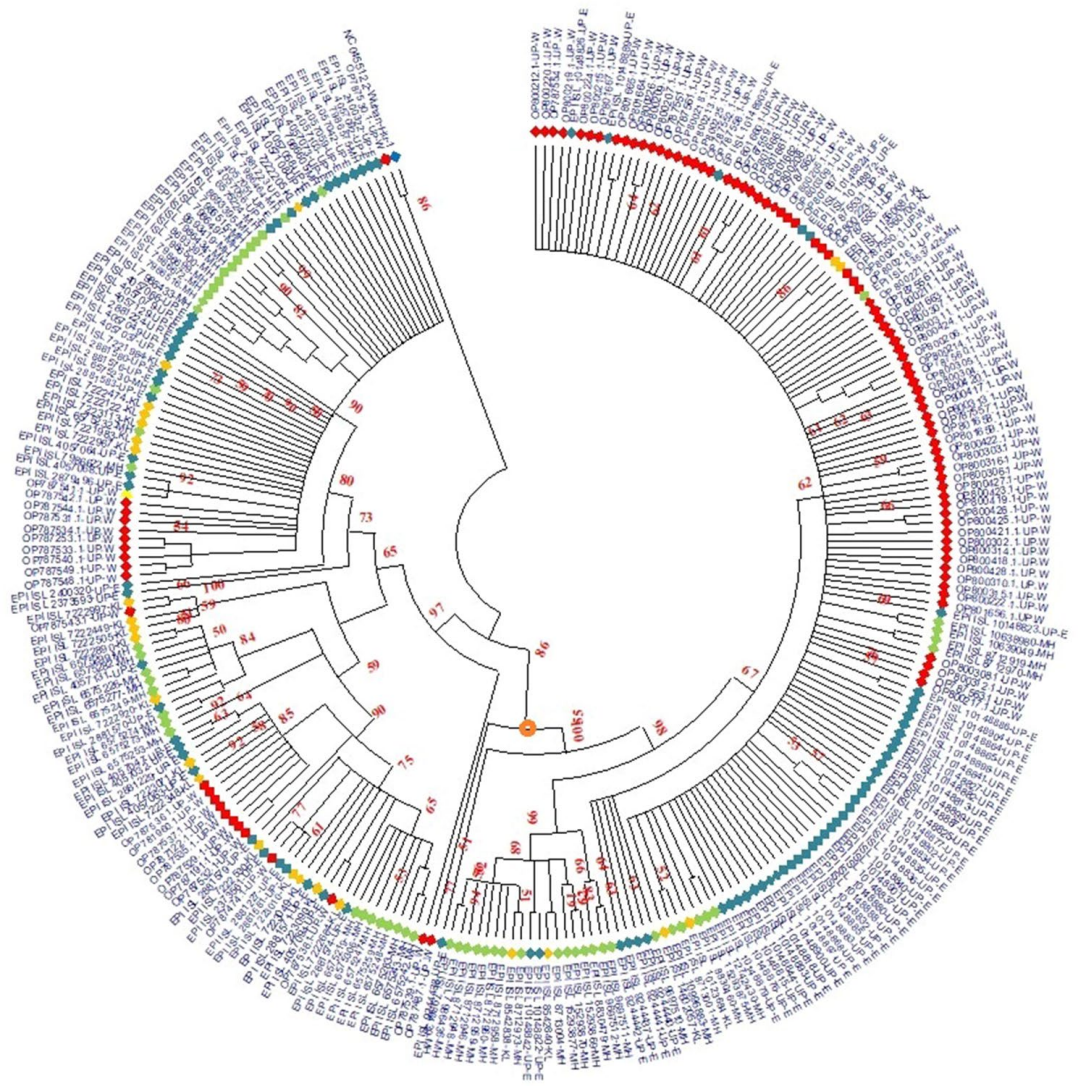
Maximum likelihood tree of the SARS-CoV-2 sequences. The maximum likelihood phylogenetic tree was inferred from the sequences retrieved from Western Uttar Pradesh (Red diamond) along with other GISAID sequences from Eastern Uttar Pradesh (light blue diamond), Maharashtra (green diamond), and Kerala (yellow diamond). The reference sequence of Wuhan is represented by (blue diamond).

Among the studied cases (99), one sequence (OP787530.1− Western Uttar Pradesh) demonstrated genetic relatedness to the Wuhan isolate having some unique variations that are not present in other isolates. A total of 270 genomes identified from different parts of India by the GISAID are categorized into four clades, viz. GRA, GK, G, and GH. Based on a genetic analysis of the Indian sequences, strains in the clade GRA comprised the largest proportion (151) followed by GK (113), GH (1), and G (1). It was noted that omicron variants from all the states (Uttar Pradesh, Maharashtra, and Kerala) have belonged to clade GRA while clade GK is majorly circulated in Delta variants from all the states. During the evolutionary development of SARS-CoV-2, the group clade designating the site of diversification (marked by a red dot) emerged as the most noteworthy (Fig. 7).

## Discussion

India was alerted about the SARS-CoV-2 transmission since the report of the first case of SARS-CoV-2 in the Kerala state^2^. The country-wide lockdown (March 2021 to January 2022) was announced by the Government of India to restrict viral transmission. A remarkable and distinct genetic diversity exists in India as evidenced by a few research studies^25,49,50^. Western Uttar Pradesh has been hit by two major COVID-19 spikes, leading to severe mortality in the state. Despite the higher number of cases during the ongoing pandemic period, there were several national movements such as the elections and many other related activities in public places that facilitated the spread of the SARS-CoV-2 infections from the cities to the rural areas^25^.

This study unravels the prevalence of VOCs such as Omicron and Delta in the Western Uttar Pradesh region. Recent studies have shown that 56 peculiar single nucleotide polymorphism (SNP) variations among SARS-CoV-2 were found in central Uttar Pradesh which was distinguished in two major clusters that showed rigorous and detrimental effects on the genome^50,51^. In the early stages of the SARS-CoV-2 pandemic, Southeast Asia (B.6.6), Europe (B.1), and other regions of India (B.1.210 and B.1.247) reported the majority of cases^32,44^. During the second spike of the pandemic in Uttar Pradesh, VOCs such as Delta and Delta AY.1 were majorly introduced^25,50,51^. The introduction of the new SARS-CoV-2 lineage B.1.617 and sub-lineage B.1.617.2 (Delta variant), a “variant of concern” in India during the second major spike of SARS-CoV-2, has led to an upsurge in consistent and breakthrough infections^51^. According to INSACOG, the diversification and grouping of seven Pangolin lineages were discovered by the SARS-CoV-2 genomic data from Uttar Pradesh. (http://clingen.igib.res.in/ covid19genomes/).

According to our study, the 99 SARS-CoV-2 samples sequenced herein, distinctively clustered to BA.2-like and B.1.617.2 Pangolin lineages respectively. The present study showed a change in the dominant clade starting from GK to GRA. Late in 2021, the Omicron variant (clade GRA) overtook the Delta variant as the dominant variant in India as well as globally^52^. Over the different epidemic waves in India, the tree branched out according to the clades and clearly demonstrated the replacement of clades over time. SARS-CoV-2 sequences generated in the lab represented some of the genetic lineages that were spreading country-wide at that time. Sequences belonging to the same Pango branch from different states were found to be more closely related. It was noted how the predominance of the various clades varied by state. Among four clades, GRA and GK showed the mixing of strains (Omicron and Delta) from Uttar Pradesh, Maharashtra, and Kerala. These genomes are clustered into two clusters of Delta and Omicron strains.

One reference sequence (OP787530.1− Western Uttar Pradesh) belongs to the 20A clade and B.1.36 Pango lineage showed very close similarity with the reference sequence of Wuhan (NC_045512.2). Phylogenetic analysis showed that SARS-CoV-2 strains (OP787530.1− Western Uttar Pradesh) could be descending from the original Wuhan strain with four unique mutations (N: S194L, ORF1a: V561A, ORF1a: P971L, ORF3a: Q57H). A previous study from China reported that Q57H mutation is responsible for the fourth wave of COVID-19 in Hong Kong China^53^. Another study from India showed that G25563T/Q57H in ORF3a (n = 190/837) was the most frequent mutation found in India, principally in Western India^54^. This reinforces the fact that travel and migration were the major contributors to the pandemic spread. In fact, the psychological fear of social isolation even prevented people from disclosing their travel history. Thus, there is a high probability that OP787530.1− Western Uttar Pradesh sequence similarity could be attributed to such potential travel/ migration history.

A characteristic mutation in the spike protein of clades GRA/GH/GK is D614G, which escalates binding to the angiotensin-converting enzyme 2 (ACE2) receptor and eventually surges the viral entry into host cells. Aside from D614G, GRA (BA.2 lineage) variants with E484A mutations also exhibit substantial antibody neutralization resistance, contributing to an improved vaccine-breakthrough capability^55^. In contrast, the B.1.617.2 lineage, which carries the mutations L452R and P681R in the spike protein, may explain the increase in cases in Western Uttar Pradesh in March 2021.

Genomic epidemiology and whole genome sequencing have been widely used to monitor the transmission and evolution of the SARS-CoV-2 virus globally^29–31^. Mutations in SARS-CoV-2 have recently emerged as a major concern around the globe, possibly affecting its transmission, infectivity, virulence, and immune escape. As of now, monoclonal antibodies and vaccines primarily target the spike protein, which plays a prime role in viral attachment and entry into host cells. The receptor-binding domain (RBD) has several genomic variations and diversity in SARS-CoV-2 spike glycoprotein^56–58^. Patient samples were collected from March 2021 to January 2022 in which most of the patients had received booster doses of the vaccine and were still infected by the virus. This indicated that there was an immunity escape observed in these patients. Three escape mutations in the S gene at codon position 19 (T19I/R), 484 (E484A/Q), and 681 (P681R/H) during the fourth and fifth waves in India have a critical role in immune escape in SARS-CoV-2 infections, were dominantly found in our study subjects. Substitution at positions P681 and E484 has become increasingly common among clinical isolates. Previous studies showed that the virulence and pathogenesis of the Delta variant could be impacted by D614G and P681R mutations. Although the D614G mutation occurs in the Omicron variant, the additional presence of the P681H mutation may result in slow cleavage. Moreover, this may limit the Omicron virus replication to the upper respiratory tract resulting in less fusion and infectivity as compared to the Delta and D614G + P681R double mutants^59^. A previous study from Brazil reported that D614G mutation was detected in 90.5% of their samples, and was recently associated with higher viral loads and increased replication on human lung epithelial cells^60^. The earlier study reported that T19R and T19I mutations in the NTD spike region were significantly associated with mortality in patients by Delta and Omicron variants, respectively^61^.

We found that L452R, T478K, E484A, N501Y, D614G, P681R, and D950N were the key mutations found in the spike protein including within the receptor-binding domain (RBD). The most common mutations (L452R, P681R, and D950N) were observed in the Delta variant (B.1.617.2 spike protein) however these mutations were absent in the Omicron variant, which is believed to be responsible for more adverse effects by the delta variant infections. In 2022, a study from china^62^ reported that the mutated Omicron-L452R was significantly more effective at infecting humanized ACE2 mice's lung tissues. A previous study also reported that RBD mutations L452R, T478K, and E484Q may possibly result in increased ACE2 binding, whereas P681R at the furin cleavage site may improve transmissibility through an increase in S1-S2 cleavage^63^. The Omicron and Delta variants were found to have a higher transmissibility rate as compared to the original strain of SARS-CoV-2 with a capability to escape the host immune response^64^ resulting in breakthrough infections. The ability of various anti-RBD-specific antibodies to bind only to the open spike protein is well recognized. Mutations that cause changes in spike glycoprotein conformation are more likely to make the RBD less susceptible to neutralizing antibodies^65–67^. A recent epidemiological and serology-based study in New Jersey revealed the presence of various mutations in the spike protein that are indicative of convergent evolution. It showed mainly L452R and T478K mapped to the Delta strain, whereas S371L, N440K, and Q493R were to the Omicron strains^68^ in line with our observations except for the presence of S371F in place of S371L.

We also found one variant OP787487.1 (B.1.633− which was globally detected in February and March 2021) harbors some specific mutation (S: L5F, S: T76I, S: D253N, S: T572N, S: A575S, S: D796H, and S: T859N) which are not present in any other virus samples. In 2022, a study^69^ also reported that in addition to Beta, Gamma, Delta, and Omicron VOCs, variant B.1.633 can induce vaccine breakthrough infections. The spike substitution mutant D796H showed decreased susceptibility to neutralizing antibodies, however, it also resulted in an infectivity defect^70^. These findings led us to believe that variation at this position might lead to a fitness cost for viral replication. These mutations which play a critical role in immune escape in SARS-CoV-2 infection, were found to be dominant in our study participants. In summary, our present study concluded that the newly emerged variants contributed to the second wave of COVID-19 in Uttar Pradesh. High-throughput sequencing makes it easier for researchers to identify and locate genetic variants of public health concerns that are useful for vaccine development allowing it to identify potential biomarkers and drug targets of COVID-19^71^. The present study is an attempt to derive a comprehensive study that highlights the pattern of circulating SARS-CoV-2 strains in Western Uttar Pradesh which comprised of the significant mutations G142D, N440K, E484A, N501Y, T478K, P681R, and D950N. These mutations played a critical role in immune escape in breakthrough infections. Additionally, the mutation D614G was coherent in most of the Pangolin which is specifically reported to be associated with viral transmissibility and high virulence. To our knowledge, this is the first study from Western Uttar Pradesh highlighting the molecular surveillance-based phylogenetic trends of whole genome sequences of SARS CoV-2. However, continuous and sustained monitoring of the identified global viral strains identified is required for an in-depth and detailed understanding of the evolution patterns of SARS-CoV-2 to explore the different evolutionary mechanisms adopted by the virus. The outcome will be immensely useful in designing a streamlined healthcare policy for Uttar Pradesh to contain any future spread of more evolved SARS-CoV-2 strains.

## Methods

### Clinical specimen collection

The National Institute of Biologicals (NIB), Noida, India is an autonomous institute under the Ministry of Health and family welfare, Government of India. It is the major testing center for SARS-CoV-2 samples as designated by the Indian Council of Medical Research (ICMR). SARS-CoV-2 suspected samples were received at NIB, Noida from various quarantine camps and hospitals located in the Western Uttar Pradesh region of India (Fig. 1) which were further processed for diagnostic testing according to the WHO guidelines^22^. During the period (March 2021 to January 2022), nasopharyngeal/oropharyngeal swabs (NPS/OPS) (n = 20,381) were collected for routine SARS-CoV-2 diagnosis. A total of 3,485 samples tested positive by real-time PCR (RT-PCR). Among 3,485 positive samples, 99 were randomly selected for sequencing. The primary inclusion criteria for the sample to be eligible for sequencing was determined by the SARS-CoV-2 positive samples that displayed a cycle threshold (Ct) of less than 30 so as to ensure maximum sequence coverage.

### Nucleic acid extraction and Real-time (RT-PCR)

Extraction of viral RNA from the suspected clinical samples was performed using QIAamp Viral RNA Mini Kit using the manufacturer’s instructions (Cat no. 52906; Qiagen, GmbH, Germany). The RT-PCR for diagnostic testing of SARS-CoV-2 nucleic acid was done using an NIV Multiplex Single Tube Real-Time PCR kit (Lot no. 11) on CFX96 Deep Well Real-time system (Bio-Rad) as per the manufacturer’s specifications.

### Next-generation sequencing

The whole genome sequencing of the selected samples was outsourced. Extracted RNA was used for the synthesis of first-strand cDNA. Commercially available PCR primers for the amplification of the complete SARS-CoV-2 genome were used for targeted enrichment. The library preparation was done and the final library distribution was evaluated on Tape Station followed by sequencing. Library preparation was done using the QIAseq DIRECT SARS-CoV2 Library Kit (Cat no.333891; Qiagen) with QIAseq DIRECT SARS-CoV-2 Enhancer (Cat no. 333884; Qiagen). Library quantification was conducted using Qubit High Sensitivity Assay. Cluster amplification on an Illumina flow cell was then achieved, followed by pooling and dilution to final optimal loading concentrations, and sequencing to produce 150 bp paired-end reads using an Illumina HiSeqX instrument (Illumina, San Diego, US).

### Phylogeny construction and analysis

Genomic sequences from Eastern UP, Maharashtra, and Kerala were obtained from the Global Initiative on Sharing All Influenza Data (GISAID)^72^ database, along with the reference sequences from this study (Western Uttar Pradesh) were used in the evolutionary analysis. In total, there were 270 sequences used to generate a cladogram (Supplementary File 1). The sequences were aligned using the MUSCLE program in MEGA software. We used the Model program in MEGA and Model Selection in IQ-TREE (http://iqtree.cibiv.univie.ac.at/) for finding the best-fit model with the lowest BIC (Bayesian Information Criterion) score. A maximum likelihood phylogenetic tree using the GTR + G + I model, was built with 1,000 bootstrap replications to assess the statistical robustness using MEGA11^73^. The tree was visualized using an online tool iToL (https://itol.embl.de/tree/115117108166307691660119911). Next clade and Pangolin COVID-19 Lineage Assigner were used for lineage/clade assignment.

### Variant identification

Using bcl2fastq v2.20 software, raw HiSeqX data was demultiplexed from binary base call (BCL) format to FASTQ format. The paired-end FASTQ reads were then preprocessed by removing low-quality bases (Q20 < 10), adapter sequences, and reads with length < 30 bp using Cutadapt (version 1.18)^74^. High-quality reads were mapped to the reference genome of SARS-CoV-2 (GenBank accession number: NC_045512.2) using the Burrows-Wheeler Aligner MEM algorithm (BWA-MEM) (version 0.7.12) using default settings for paired-end mode^75^. The SAM tools package^76^ was used to retain reads with high mapping quality (MQ > 25), and the Mark Duplicates package was used to identify duplicate reads in the Genome Analysis Toolkit (GATK v4.1.0.0)^77^. Further, the genomic variants were predicted using uniquely-mapped reads by the GATK Haplotype Caller package. Genotypes were assigned to mutants (mutant allele frequency ≥ 0.7), degenerate nucleotides (mutant allele frequency < 0.7 and ≥ 0.3), and reference alleles (mutant allele frequency < 0.3). Further variations were also identified using the Nextclade web server (https://clades.nextstrain.org/). Ensembl Variant Effect Predictor (VEP) was used to annotate the impact of variants on genes and protein sequences^78^.

### Ethical statement

The National Institute of Biologicals (NIB), Noida is an apex autonomous institute under the administrative control of the Ministry of Health and Family Welfare (MoHFW), Government of India. NIB is not a hospital-based institution, however, it has been entrusted to perform COVID-19 testing since March 2020 during the pandemic. The present study has been performed using the leftover, anonymized COVID-19 samples, wherein all the methods and protocols were in concordance with the standard guidelines and regulations. All the experimental protocols carried out in the current study were approved by the Director of NIB, Noida.

### Supplementary Information

Supplementary Information.

## Data Availability

The methodology and original data of the present study are included in the article. Complete genome sequences generated from this study have been submitted to NCBI. The Bio Project accession ID is PRJNA976493 and the respective BioSample accession numbers are SAMN35370730-SAMN35370828. Additionally, the SARS-CoV-2 whole genome sequences are available in the GenBank repository in released form, and the subsequent accession IDs have been included in Supplementary File 3. Additional information has been provided in Supplementary files 4, 5.
