# Author Correction: Genomic epidemiology of SARS-CoV-2 from Uttar Pradesh, India

**DOI:** 10.1038/s41598-023-43636-3

**Published:** 2023-10-04

**Authors:** Gauri Misra, Ashrat Manzoor, Meenu Chopra, Archana Upadhyay, Amit Katiyar, Brij Bhushan, Anup Anvikar

**Affiliations:** 1 Molecular Diagnostics and COVID-19 Kit Testing Laboratory, National Institute of Biologicals (Ministry of Health and Family Welfare), A-32, Sector-62, Institutional Area, Noida, UP 201309 India; 2 National Dairy Research Institute, Karnal, Haryana India; 3 Bioinformatics Facility, Centralized Core Research Facility, All India Institute of Medical Sciences, Ansari Nagar, New Delhi, 110029 India

Correction to: *Scientific Reports* 10.1038/s41598-023-42065-6, published online 08 September 2023

In the original version of this Article Gauri Misra was incorrectly affiliated with ‘Molecular Diagnostics and COVD-19 Kit Testing Laboratory, Ministry of Health and Family Welfare, Noida, U.P., 201309, India’. Additionally, Affiliation 1 was incorrectly stated as ‘A-32, Sector-62, Institutional Area, National Institute of Biologicals, Noida, UP, 201309, India.’ The correct Affiliation 1 is listed below.

Molecular Diagnostics and COVID-19 Kit Testing Laboratory, National Institute of Biologicals (Ministry of Health and Family Welfare), A-32, Sector-62, Institutional Area, Noida, UP, 201309, India.

The original Article has been corrected.

